# Oral immunotherapy for the treatment of pumpkin seed allergy: a real-world case series

**DOI:** 10.1186/s13223-026-01043-z

**Published:** 2026-06-02

**Authors:** Victor Paradis, Meghan Kanou, Louis Paradis, Roxane Labrosse, Kathryn Samaan, Philippe Bégin, Florence Gingras-Lessard, François Graham, Anne Des Roches

**Affiliations:** 1 Division of Clinical Immunology and Allergy, Department of Pediatrics, CHU Sainte-Justine, Université de Montréal, Montreal, Qc Canada; 2 Division of Clinical Immunology and Allergy, Department of Medicine, CHUM, Université de Montréal, Montréal, Qc Canada

**Keywords:** Pumpkin seed, Oral immunotherapy, Allergy, Desensitization

## Abstract

**Background:**

Pumpkin seed, a member of the Cucurbitaceae family, is increasingly consumed because of its high protein content and perceived health benefits. Along with its growing use, cases of pumpkin seed allergy are being reported. However, data on pumpkin seed allergy and oral immunotherapy (OIT) remain scarce.

**Methods:**

We conducted a retrospective chart review at a tertiary pediatric center (Sainte-Justine University Hospital Center, Montreal, Canada) including all patients who initiated or completed pumpkin seed OIT since 2019. OIT protocols were individualized, with dose increases typically performed every four weeks. Target maintenance doses were at least 300 mg of pumpkin seed protein.

**Results:**

Eleven patients (median age at OIT initiation: 6.5 years; range 1–12) underwent pumpkin seed OIT. Ten patients (91%) reached maintenance dosing within a median of 9.5 months (range 6–22) while one patient discontinued OIT due to persistent abdominal pain. No anaphylactic reactions occurred at home during treatment. Two anaphylactic reactions requiring epinephrine occurred during in-clinic up-dosing visits in a single patient, despite this, the patient ultimately achieved maintenance. Gastrointestinal and oral symptoms were the most frequent adverse events and were generally managed with temporary premedication. Most patients (91%) underwent concomitant multi-food OIT. Patients with significant adverse reactions had high ratios of pumpkin-specific IgE to total IgE.

**Conclusion:**

Pumpkin seed OIT appears feasible in a highly atopic pediatric population, with a safety profile comparable to that reported for OIT to other food allergens. Given the increasing dietary exposure to pumpkin seeds, larger prospective studies are needed to better define risk factors and long-term outcomes.

## Background

Pumpkin seed is an emerging allergen belonging to the Cucurbitaceae family [1]. Over the last decade, its consumption has increased substantially due to its widespread incorporation into breads, snacks, salads and plant-based dietary products [2]. Pumpkin seeds are valued for their nutritional benefits, including omega-3 and omega-6 fatty acids [1].

While allergies to other edible seeds such as sesame, mustard, and sunflower are well characterized, pumpkin seed allergy remains poorly described, with only isolated case reports and small series published to date [1, 3]. However, pumpkin seed allergy may be associated with severe reactions, including anaphylaxis, and shows significant rates of co-sensitization and co-allergy with other seeds, particularly sesame and sunflower [3].

Oral immunotherapy (OIT) has emerged as an effective disease-modifying treatment for several food allergies, including peanut, milk, egg, and sesame, with increasing evidence supporting its feasibility and safety in real-world clinical settings [4]. Given that allergies to seeds and nuts are generally associated with low rates of spontaneous remission, making natural resolution over time unlikely for most affected individuals, there is a strong rationale for pursuing active treatment with OIT rather than expectant management based on an elimination diet [5–7]. Despite these advances, no published data are currently available regarding OIT for pumpkin seed allergy.

The objective of this case-series was to describe our real-world experience with pumpkin seed OIT in a pediatric population, focusing on feasibility, safety, and clinical outcomes.

## Methods

### Study design and population

We conducted a retrospective chart review at Sainte-Justine University Hospital Center (Montreal, Canada). All pediatric patients who initiated pumpkin seed oral immunotherapy (OIT) between January 2019 and December 2023 were eligible for inclusion. All patients had a confirmed diagnosis of pumpkin seed allergy based on a convincing clinical history of IgE-mediated reaction, as assessed by an allergist, and evidence of sensitization, including positive skin prick testing and/or detectable pumpkin seed–specific IgE. As this was a real-world study, oral food challenges (OFC) were not routinely performed. Patients undergoing concomitant multi-food OIT were not excluded. No formal exclusion criteria were applied beyond standard contraindications to OIT, including uncontrolled asthma or suspected eosinophilic gastrointestinal disease.

### Oral immunotherapy protocol

Pumpkin seed OIT was conducted using a non-commercial, food-based protocol. Pumpkin seed powder (Yupik®) was used for initial dosing, with transition to pumpkin seed butter (Nuts to You® or Yupik®) once higher doses were tolerated. Initial dose, escalation schedule, and target maintenance dose were individualized based on patient characteristics and family preferences.

Dose escalations were performed during supervised clinic visits, typically every four weeks. Patients were observed for at least one hour following each up-dosing visit. Adverse reactions occurring at home were prospectively documented using a standardized symptom diary and reviewed at each clinic visit, while reactions during up-dosing visits were recorded by the treating physician. High-dose oral food challenges up to four times the daily dose were performed to assess the level of desensitization while on maintenance It was offered to families when specific IgE and/or skin prick test had decreased by at least 50% or after 5 years. Patients passing the high-dose OFC were allowed to decrease dosing to three times a week and introduce pumpkin seed in the diet ad lib. Sustained unresponsiveness (OFC after a period of avoidance) was not tested in this cohort.

### Outcomes

Successful OIT was defined as the ability to reach a dose of 300 mg of pumpkin seed protein without ongoing dose-limiting adverse reactions. Occurrence of adverse reactions, including anaphylaxis requiring epinephrine are reported as safety outcomes. Other outcomes included time to a dose of 300 mg, need for premedication, and outcome of high-dose OFC when performed.

### Ethics

This study was approved by the Sainte-Justine Research Ethics Board.

## Results

Twelve pediatric patients (median age 6.5 years; range 1–12) underwent pumpkin seed OIT during the study period. One patient did not have a history of clinical reaction to pumpkin seed and was therefore excluded from the study. Baseline clinical characteristics are summarized in Table 1 and OIT related data are presented in Table 2. Individual patient data are available in Table 3.

**Table 1:** Characteristics of the population

	N = 11
Age at reaction, y (median, min/max)	5(1 -11)
Age at OIT, y (median, min/max)	7 (1—12)
Male sex (%)	5 (42%)
Comorbidities	–
Atopic dermatitis	9(82%)
Asthma	6 (55%)
Allergic rhinitis	3 (27%)
Other food allergy	11(100%)
Number of other food allergy (median, min/max)	5 (1–13)
Seed allergy	8 (73%)
Sesame	6 (55%)
Mustard	2(18%)
Sunflower	0(0%)
Peanut	6 (55%)
Milk	4 (36%)
Egg	5 (45%)
Nuts	8 (73%)
Wheat	1 (9%)
Other	4 (36%)
Food sensitization to seed	11(100%)
Sesame	8(73%)
Mustard	2 (18%)
Sunflower	3(27%)
Environmental sensitization	7 (64%)
Birch	4/7 (57%)
Grass	4/7 (57%)
Ragweed	2/7 (29%)
Mugwort	3/7 (43%)
Dust mites	3/7 (43%)
Coackroach	2/7 (29%)
Cat/dog danders	4/7 (57%)

**Table 2:** Description of pumpkin OIT course and allergic characteristics of the population

	N = 11
Age at OIT, y (median, min/max)	7 (1—12)
Worst pumpkin reaction before OIT	–
CoFAR 1	5 (45%)
CoFAR 2	4 (36%)
CoFAR 3	2 (18%)
Skin test to pumpkin, mm (median, min/max)	10 (4 -20)
Total IgE, kU/L (median, min/max)	761 (71–4959)
Specific IgE to pumpkin, kU/L (median, min/max)	11 (0,8- over 100)
Ratio Specific IgE / Total IgE (%)	2 (1—over 50%)
OIT to multiple allergens (%)	10 (91%)
Maintenance dose reached	10(91%)
OIT stopped	1 (9%)
Time to maintenance dose (month)	9,5 (6–22)
High dose challenge tolerated	5 (45%)
Allergic reaction during OIT	9 (82%)
Anaphylaxis	1 (9%)
Gastrointestinal	4 (36%)
Respiratory	1 (9%)
Oral	5 (45%)
Skin	2 (18%)
Epinephrine during OIT	1 (8%)

**Table 3:** Cases description

Case	Sex	Age at OIT	Skin test to pumpkin (mm)	Specific pumpkin IgE (kU/L)	Total IgE (kU/L)	Ratio specific IgE /Total IgE (%)	Other allergen(s) under simultaneous OIT	Starting allergen dose (mg)	Allergen dose at maintenance (mg)	Time to reach maintenance dose (month)
1	M	11	11	> 100	761	> 50	Sesame, peanut	3	OIT stopped	–
2	F	6	11	5,69	776	0,7	Milk, egg, cashew, pistachio, mustard	100	300	2*
3	M	6	4	> 100	204	> 50	Sesame	6	666	6
4	M	1	4	3,75	1220	0,3	Egg, wheat, peanut, almond, cashew	33	900	8
5	M	9	11	11,7	792	1,4	Sesame, peanut, cashew	3	1666	7
6	M	12	5	10,8	385	2,8	Cashew, pistachio	15	1000	10
7	F	3	10	77	4959	1.6	Kiwi	5	416	7
8	F	11	4	0,77	71,5	1	None	5	333	7
9	F	9	10	134	3820	3.6	Sunflower, Brazil nut, chickpea, lentil	3	412	15
10	F	7	20	6,07	179	3,4	Peanut	5	316	10
11	M	3	4	–	137	–	Milk, egg, sesame, cashew, walnut	1	800	18

^*^OIT under omalizumab

The delay between the initial reaction and OIT initiation was 8 months (range: 3–120). Patients with longer delays had experienced anaphylaxis and had IgE levels > 10 kU/L and/or skin prick test wheals > 10 mm at the time of OIT initiation.

The cohort was highly atopic, with atopic dermatitis present in 82%, asthma in 55%, and allergic rhinitis in 27%. All patients had at least one additional food allergy, with a median of five food allergies per patient (range 1–13).

Baseline testing showed a median pumpkin seed skin prick test of 10 mm (range 4–20 mm), median specific IgE of 11 kU/L (range 0.8 to > 100 kU/L), and median total IgE of 761 kU/L (range 71–4959 kU/L).

Up-dosing visits occurred approximately every four weeks (median interval 4 weeks; range 1–16), with a median dose increase of 100% per visit (range 25–100%). The target maintenance dose was determined in collaboration with each family, reflecting share-decision making, and ranged from 300 to 1666 mg of pumpkin seed protein (median of 540 mg). For OIT initiated after 2022, the median maintenance dose was 412 mg (range from 316 to 800 mg).

The maintenance dose was achieved after a median of 9 months (range 6–22), except for one patient who discontinued OIT due to persistent abdominal pain. This patient had a severe pumpkin seed allergy, with pumpkin seed-specific IgE > 100 kU/L and a specific IgE-to-total IgE ratio greater than 50%.

Four patients underwent high dose OFC after one, two, two and five years of OIT, respectively, after tests had decreased by 50%. A fifth patient underwent high-dose OFC after 5 years despite allergy tests remaining the same. All patient passed their OFC and subsequently introduced pumpkin seed in their diet ad lib and decreased dosing to three times a week. The remaining six patients continue on maintenance dosing.

Ten of the eleven patients underwent concomitant multi-food OIT. One patient received omalizumab during treatment. No episode of home anaphylaxis occurred.

### Safety and adverse reactions

Two anaphylactic reactions requiring epinephrine occurred during in-clinic up-dosing visits in a single patient undergoing multi-food OIT (pumpkin seed and sesame), with specific IgE levels > 100 ku/L and 1.34 kU/L, respectively, suggesting the pumpkin seed as the most likely trigger. Despite these reactions, the patient successfully reached a maintenance dose of 833 mg.

Three additional patients required premedication with antihistamines and/or sodium cromoglycate to manage gastrointestinal discomfort during the up-dosing phase, including the patient who ultimately discontinued pumpkin seed OIT. This patient was undergoing OIT for pumpkin seed, sesame, and peanut, with specific IgE levels > 100, 2, and 0.79 kU/L, respectively. Despite discontinuation of pumpkin seed from his OIT, the patient continued to experience abdominal pain. An upper gastrointestinal endoscopy was performed and showed normal findings. Gastrointestinal symptoms persisted even after discontinuation of OIT to the other allergens, suggesting a possible functional etiology rather than an adverse effect of OIT.

One patient required antihistamine premedication to control oral pruritus. Except for the patient who discontinued pumpkin seed OIT, all patients were able to stop premedication at maintenance.

### Co-sensitization and co-allergy

The rate of co-sensitization with other seeds was 81%, most commonly to sesame (73%), followed by sunflower (27%) and with mustard (18%). The rate of confirmed seed co-allergy was 73%, primarily to sesame (55%), and less frequently to mustard (18%).

## Discussion

This is, to our knowledge, the first published case series describing OIT for pumpkin seed allergy. Our real-world experience suggests that pumpkin seed OIT is feasible and associated with an acceptable safety profile, comparable to that reported for other food allergens [4].

Pumpkin seed allergy remains poorly described in the literature, despite increasing dietary exposure related to plant-based diets and the use of seeds as nut alternatives. The higher rate of sesame co-sensitization in our cohort may reflect referral bias toward a more severely allergic population in a tertiary care center [3]. Given the documented rates of co-sensitization and co-allergy with other seeds, particularly sesame and sunflower, an increase in pumpkin seed allergy can reasonably be anticipated.

Our cohort represents a highly atopic population, which may partly explain the frequency of adverse reactions observed during OIT. However, the rate and nature of these reactions were similar to those reported in OIT studies for other foods [4]. Notably, the two patients who experienced significant adverse outcomes—persistent gastrointestinal symptoms leading to discontinuation and anaphylaxis requiring epinephrine—both had very high ratios of specific IgE-to-total IgE. This observation supports previous findings suggesting that the specific-to-total IgE ratio may be a useful prognostic marker for OIT outcomes [8]. This is further illustrated by patients with high pumpkin seed-specific IgE but low specific-to-total IgE ratios, who tolerated OIT without severe reactions (Table 3).

This study has several limitations, including its retrospective design, small sample size, and the frequent concomitant multi-food OIT, which may limit attribution of adverse events. In addition, long-term outcomes such as sustained unresponsiveness were not assessed.

One limitation of this study was that OFC were not systematically performed prior to OIT, which leads to uncertainty regarding baseline clinical reactivity. We cannot be certain that patients were not already able to tolerate 300 mg of pumpkin seed protein prior to OIT. This study was not intended to demonstrate efficacy but rather demonstrate feasibility in a real-life setting. All participants had a convincing history of clinical reaction to pumpkin seed as well as positive skin test and serum specific IgE. While almost all participants experienced reactions to their OIT mix, we cannot confidently attribute these reactions to pumpkin seed, as most participants were desensitized to more than one food.

The target maintenance dose varied across our sample. This reflects a change in practice over time, as the target maintenance dose decreased following peanut OIT studies showing the lack of superiority of high maintenance doses [9]. Moreover, individual maintenance doses were influenced by parental preferences, including taste aversion and convenience. This said all patients were able to consistently achieve a maintenance dose of at least 300 mg pumpkin seed protein.

All five patients who underwent high-dose OFC were followed for a median of 32 months (min–max: 10–54). All continue to consume pumpkin seed regularly and remain tolerant. Sustained unresponsiveness (SU) was not assessed and we cannot draw any conclusion regarding the efficacy of pumpkin seed OIT at inducing clinical remission. All patients chose to undergo treatment with the understanding that it would involve continuous dosing with no guarantee of ever reaching SU.

Despite these limitations, this real-world case series provides novel and clinically relevant data on an emerging food allergen. As pumpkin seed becomes increasingly prevalent in the diet, greater awareness of its allergenic potential and of available therapeutic options such as OIT will be essential.

## Data Availability

Data is provided within the manuscript or supplementary information files.

## Abbreviation

OIT: Oral immunotherapy
